# An International Comparison of Web-based Reporting About Health Care Quality: Content Analysis

**DOI:** 10.2196/jmir.1191

**Published:** 2010-04-13

**Authors:** Olga C Damman, Ylva KA van den Hengel, A Jeanne M van Loon, Jany Rademakers

**Affiliations:** ^2^RIVM (National Institute for Public Health and the Environment)Bilthoventhe Netherlands; ^1^NIVEL (Netherlands Institute for Health Services Research)Utrechtthe Netherlands

**Keywords:** Consumer health information, information display, decision making, Internet, international comparison, content analysis

## Abstract

**Background:**

On more and more websites, consumers are provided with public reports about health care. This move toward provision of more comparative information has resulted in different information types being published that often contain contradictory information.

**Objective:**

The objective was to assess the current state of the art in the presentation of online comparative health care information and to compare how the integration of different information types is dealt with on websites. The content analysis was performed in order to provide website managers and Internet researchers with a resource of knowledge about presentation formats being applied internationally.

**Methods:**

A Web search was used to identify websites that contained comparative health care information. The websites were systematically examined to assess how three different types of information (provider characteristics and services, performance indicators, and health care user experience) were presented to consumers. Furthermore, a short survey was disseminated to the reviewed websites to assess how the presentation formats were selected.

**Results:**

We reviewed 42 websites from the following countries: Australia, Canada, Denmark, Germany, Ireland, the Netherlands, Norway, the United Kingdom, the United States, and Sweden. We found the most common ways to integrate different information types were the two extreme options: no integration at all (on 36% of the websites) and high levels of integration in single tables on 41% of the websites). Nearly 70% of the websites offered drill down paths to more detailed information. Diverse presentation approaches were used to display comparative health care information on the Internet. Numbers were used on the majority of websites (88%) to display comparative information.

**Conclusions:**

Currently, approaches to the presentation of comparative health care information do not seem to be systematically selected. It seems important, however, that website managers become aware of the complexities inherent in comparative information when they release information on the Web. Important complexities to pay attention to are the use of numbers, the display of contradictory information, and the extent of variation among attributes and attribute levels. As for the integration of different information types, it remains unclear which presentation approaches are preferable. Our study provides a good starting point for Internet research to further address the question of how different types of information can be more effectively presented to consumers.

## Introduction

Public reporting of comparative health care information has become increasingly important in several countries. Comparative health care information is information by which consumers can make explicit comparisons between the performances of health care providers or health plans in order to make an informed choice. In the United States and the United Kingdom, efforts to make this kind of information publicly available have been ongoing for about fifteen years. The aims are to increase public accountability and to support consumer choice in health care and indirectly to improve the quality of health services. Health care policy in the Netherlands currently focuses on transparency as well: health care consumers are encouraged to make use of public comparative information about health care services and quality [1,2]. Dutch consumers have been provided with public reports of health care information in newspapers and magazines since the late 1990s. In addition, comparative health care information has been published on the Internet in the Netherlands for the past few years.

The number of websites containing comparative health care reports is rapidly growing worldwide. This number will continue to rise given the increased tendency of many health care systems to become publicly accountable and to use market-based approaches. In addition, existing websites likely will offer more types of information as well as information about different health care sectors in order to support health care consumers’ decision making. When we look at various websites, no standard approaches for presenting information seem to emerge. Carlisle [3] examined ten American websites and concluded that “each is unique in presentation of grades and how the grades are tabulated.” However, based on laboratory studies on human decision making, it is known that information presentation formats influence consumers’ responses [4,5]. Therefore, it is necessary to reflect on and learn from the presentation approaches used in different countries within the rapidly growing movement of public health care reporting.

In fact, presentation formats of comparative health care information have been steadily gaining attention. Poor information presentation is frequently cited in the literature as one reason that this kind of information is rarely used by consumers [6-8]. Despite several years of international experience, there is little evidence that health care reports support consumer decision making [9-11]. Many researchers have suggested that the information presented is too complex for consumers and is not adjusted to consumers’ cognitive processing and decision making strategies. In a recent review, Fung and colleagues [8] concluded that “[d]espite its theoretical appeal, making public reporting work requires successfully addressing several challenges, most notably designing and implementing a reporting system appropriate for its purpose.”

One of the difficulties that consumers may face is the large amount of information on Web pages, which is often overwhelming [12]. It is known that consumers can only process a few “chunks” of information simultaneously [13] and are easily overloaded by information [14,15]. Consumers’ attitudes toward the amount of information on websites are somewhat mixed: higher numbers of features on websites have been associated with both positive [16,17] and negative [18] attitudes. In this context, the number of different types of information and the structure in which these are presented are important [19].

With respect to public comparative health care information, van Loon and Tolboom [20] defined three different information types. The first type is information about the characteristics and services provided by individual health care providers and health care facilities. This is factual information about providers’ names, addresses, and the geographic region in which health care is provided as well as information about the type of provider (eg, academic or non-academic hospital), provider specialty, available facilities, provider’s religion, costs of services, and waiting times. The second information type is information about quality of health care based on performance indicators, usually derived from existing provider registrations (ie, administrative records) or registrations required by governments and established for public reporting purposes. These concern medical and health care performance information based on relatively factual information relating to a particular health facility such as the number of patients with pressure wounds or the number of operations of a particular type. The third information type is quality information based on health care user experience. Like the second information type, this information type concerns health care performance. However, in this case, the data are derived from patient surveys. For example, patients or clients are surveyed about their experiences with the treatment in the hospital or about their satisfaction with the food or privacy in the nursing home. Within each of the three types of information, several subtypes can be distinguished as well, such as general quality indicators and more specific underlying aspects of care.

Using different information types and various indicators to make a decision is known to be a difficult cognitive process [5,21]. Moreover, as the amount of information on a Web page increases, a simple information structure combined with high usability is, almost inevitably, not attainable. Apart from the fact that more information types will increase the amount of comparative information, presenting different information types can be complex in itself. For example, it is a complex task for consumers to make a choice when a health care provider performs well on one specific quality aspect but badly on another. It can become even more complex when indicators stemming from different information types are contradictory although they concern the same aspects of care. This can be the case when quality information is drawn from both hospitals’ administrative records and patient surveys. A hospital’s registration may indicate, for example, that patients have the opportunity to participate in the decision for a particular type of anesthetic. This would be reflected by the score “yes” on the quality indicator “patient participation in choice of anesthetic.” Despite this, results of a patient survey may show that patients reported negative experiences concerning participation in decision making. For example, if patients at a particular hospital were more negative compared with patients at other hospitals, the first hospital’s performance would be given a lower rating. The question is how consumers are supposed to deal with these kinds of complexities. We know that consumers may respond differently to information depending on its complexity [22-24]. For example, the number of contradictions in the information increases information complexity, which can affect decision making accuracy [24].

An additional difficulty might emerge when different information types are presented by different information displays, such as numbers versus stars. It is unknown whether inconsistent information displays further hamper consumers’ ability to process comparative health care information.

Information display, such as words, numbers and symbols, may be another source of difficulty for consumers. In an early review of McCormack and colleagues [25], concerned largely with offline comparative health care information, the dominant presentation approaches consisted of combinations of text and graphics or text and percentages in a table format. The use of numbers may lead to confusing and overwhelming information display. Consumers may not have an emotional or affective understanding of numbers and the information may therefore be less “evaluable,” a term coined by Hibbard to refer to the ease or precision with which the values of the attributes across alternatives create an affective (good/bad) feeling [21,27]. Hibbard and colleagues demonstrated that visual display in the form of stars facilitated consumers’ comprehension and use of comparative health care information [26,27]. Previous research has also shown that the readability of text is important for consumers [28,29], and that health information on the Internet is often beyond consumers’ reading ability [30,31].

In short, the large amount and variety of information as well as how the information is presented are important issues pertaining to the publication of comparative health care information. Indeed, these issues have been cited in the literature since the early days of offline and online public reporting [3,25]. In the past decade, the number of different information types has increased, largely due to emerging information technology. Apart from a few reviews of information types presented and presentation formats used [3,19,25], no comprehensive reviews have been conducted recently. More importantly, since more countries are adopting a public reporting system for health care information, it is of interest to document which strategies are applied in countries outside the United States to present different types of health care information. If we want to understand the decision maker’s current health care information environment and be able to simplify it, an up-to-date overview of what consumers are actually confronted with is needed.

The aim of the present paper was to describe how different types of information are presented on websites containing public comparative health care information. Our primary concern was the structure used to integrate different information types. We further reviewed the drill down paths offered on websites and how information was displayed. Drill down paths are paths that provide options to get more detailed information that may also be used to structure the total amount of available information. Information display can make information more valuable to consumers. Our intention was not to review all of the websites that exist worldwide but rather to provide an overview of the state of the art that can be used as a resource of knowledge for website managers and Internet researchers. Our research question was: “How are different types of Web-based comparative health care information presented worldwide?”

## Methods

### Search Strategy

This study was conducted from July to September 2008. Two key Dutch publications on public reporting of health care were used as a starting point to search for websites providing health care information to the public [32,33]. These studies only included countries in which both performance indicators and public reporting have been incorporated in the health care system. We then performed a search using the Web search engine Google for particular terms and their translations in English, German, French, Spanish, Italian, Dutch, Norwegian, Swedish, Danish, and Finnish. The terms chosen were: quality, quality indicators, health care, compare, choose, information, patients, consumers, satisfaction, health plans, hospitals, nursing homes, home care, and mental health care. We included only websites that contained comparative information, that is, information by which consumers can make explicit comparisons between health care providers or health plans. For websites where information for health care providers was presented separately, we reviewed only the comparative information. We chose to do this because, as stated previously, comparative information is intended to facilitate consumer choice in health care.

### Analyses

We visited the selected websites and assessed the presentation approaches that were used. The following aspects were systematically considered: (1) the health care sector(s) for which information was presented; (2) the types of information presented; (3) the degree of integration of different information types; (4) the drill down paths provided; and (5) the information displays used.

For types of information, we followed the classification system of Van Loon and Tolboom [20] for public health care information: “A” indicated factual information based on provider characteristics and services; “B” indicated quality information based on performance indicators; and “C” indicated quality information based on health care user experience. The degree to which different information types were displayed in an integrated way was also assessed. In the absence of a ready taxonomy of classifying presentation formats, we classified information integration as: “0” to mean no integration, that is, different information types on different pages; “1” to mean limited integration, that is, different types of information on one page, but no integration in a single table; “2” to mean a medium amount of integration, that is, different information types on one page but clearly separated from each other; and “3” to mean a high level of integration, that is, different information types were presented in a single table. Drill down paths were assessed qualitatively according to the different approaches on the websites; we used no particular classification system. Finally, we reviewed the display of information and focused on the use of words, numbers, bar graphs, and different types of symbols. All analyses and coding activities were performed by two of the authors (OD and YH) independently. They discussed their findings and searched for agreement.

Besides reviewing the website content, we disseminated a short survey to each website included in this study. This survey contained open and closed questions about which types of information the website presented and how the presentation formats were chosen. The survey was either directly mailed to the website (in case a direct contact address was found on the website) or delivered indirectly by contacting the website through a request form. Respondents could return the completed survey to the researchers by email or by post.

## Results

### Search Results

In total, we found 42 websites in 10 different countries that presented comparative health care information. Table 1 gives a short description of each website. Most websites we identified were in the United States, although we also found a range of websites in the United Kingdom, Germany, and the Netherlands. The aim of most reporting systems was to inform consumers about health care performance and to support consumers’ choices. A few websites were not explicitly designed for consumers, but because these websites were intended to increase public accountability and were accessible for consumers, we included them in the current study.

**Table 1:** Brief descriptions of reviewed websites

Country and Website^a^		URL (Archived WebCite URL)^b^	Description


**Australia**			
	1. Your Hospitals	http://www.health.vic.gov.au/yourhospitals (http://www.webcitation.org/5clVd3AEQ)	Initiative of the Consumer Participation and Information Program. The aim is to provide information to patients, caregivers, and health care professionals. The information is generated by the Department of Health, its funded agencies, and special interest groups.

**Canada**			
	2. Hospital Report	http://www.hospitalreport.ca (http://www.webcitation.org/5clVfMnoX)	Initiative of the HHRC (Hospital Report Research Collaborative). The aims are to increase public accountability and to improve quality of care.

**Denmark**			
	3. Sundhed	http://www.sundhed.dk (http://www.webcitation.org/5clVsUhI3)	Initiative of the Danish Ministry of Health. The reporting system ‘Sundhedkvalitet’ is managed by the National Board of Health. The aim is to support consumers in their health care choices.

**Germany**			
	4. Weisse Liste	http://www.weisse-liste.de (http://www.webcitation.org/5clW384wA)	Initiative of the Bertelsmann Stiftung in collaboration with patient associations and scientific partners. The aims are to empower consumers and to support them in their health care choices.
	5. Klinik Führer Rhein-Ruhr	http://www.kliniken-rhein-ruhr.de (http://www.webcitation.org/5clW4zeOf)	Initiative of the Initiativkreis Ruhrgebiet Verwaltungs-GmbH (a collaborative of hospitals) in collaboration with scientific partners. The aim is to support consumers in their health care choices. The information is generated from the hospitals and from patient surveys.
	6. Klinikführer Rheinland	http://www.klinikfuehrer-rheinland.de (http://www.webcitation.org/5clWCp5WX)	Initiative of the Krankenhauszweckverband Köln, Bonn, und Region (KHVZ) (a collaborative of hospitals). The aim is to support consumers in their health care choices. The information is generated from the hospitals by the KHVZ.
	7. Hamburger Krankenhaus-spiegel	http://www.hamburger-krankenhausspiegel.de (http://www.webcitation.org/5clWR10vD)	Initiative of 25 hospitals in collaboration with other partners. The aim is to support consumers in their health care choices, and to stimulate providers’ quality improvement initiatives. The information is generated from the hospitals by independent audit parties.
	8.Klinikbe- wertungen	http://www.klinikbewertungen.de (http://www.webcitation.org/5clWVbRiG)	Initiative of MedizInfo, which is an Internetportal about health and health care. The aim is to provide an independent online forum about consumers’ experiences in order to help consumers in their health care choices. A second aim is to stimulate providers’ quality improvement initiatives. The information is generated from consumers’ reports on the forum.

**Ireland**			
	9. Health Information and Quality Authority	http://www.hiqa.ie (http://www.webcitation.org/5clZNb8km)	Initiative of the Health Information and Quality Authority (part of the government’s health reform program). The aims are to monitor quality of care on a set of standards and to stimulate improvement initiatives. A third aim is to help consumers in their health care choices.

**The Netherlands**			
	10. kiesBeter	http://www.kiesBeter.nl (http://www.webcitation.org/5clT0whdn)	Initiative of the Ministry of Health and managed by the National Institute for Public Health and the Environment (RIVM) in collaboration with patient associations, health care providers, and scientific partners. The aim is to provide an independent portal for all questions from the public about health and health care. One particular aim is to support consumers in their health care choices.
	11. Independer Gezondheids-zorg	http://www.independer.nl (http://www.webcitation.org/5clU1lwM3)	Initiative of Independer.nl in collaboration with other parties. The aim is to increase transparency and to support consumers in their health care choices. The information is generated by the external parties, Mediquest and Zorgweb.
	12. Zorgkiezer	http://www.zorgkiezer.nl (http://www.webcitation.org/5clU6C5MZ)	Initiative of DGN Publishers (Internet company) in collaboration with health care providers and health insurance companies. The aim is to help consumers and health care professionals in their choices. The information is generated by the website editors.
	13. Zorgbelang	http://www.zorgbelang-nederland.nl (http://www.webcitation.org/5clUFSXlV)	Initiative of Zorgbelang Nederland (association of local organizations advocating health care consumers’ interests) in collaboration with patient associations and other parties. The aim is to provide the public with information about health care.
	14. Agis Zorggids	http://www.agisweb.nl (http://www.webcitation.org/5clUPVzGY)	Initiative of health insurer Agis. The aim is to inform the insured about their options in health care (concerning contracted providers) and to provide public accountability for the activities of Agis. The information is generated by external parties.
	15. Menzis behandelwijzer	http://www.menzis.nl (http://www.webcitation.org/5clUWhkfA)	Initiative of health insurer Menzis. The aim is to support the insured in their health care choices (concerning contracted providers). The information is generated by the health purchase department and by external parties.
	16. VGZ Zorggids -Vergelijk en kies	http://www.vgz.nl (http://www.webcitation.org/5clUcadC0)	Initiative of health insurer VGZ. The aim is to support the insured in their health care choices (concerning contracted providers). The information is generated by external parties.
	17. CZ Ziekenhuisver-gelijker	http://www.cz.nl (http://www.webcitation.org/5clUhnHi0)	Initiative of health insurer CZ. The aim is to support the insured in their health care choices (concerning contracted providers). The information is generated by external parties.
	18. AD Ziekenhuisver-gelijker	http://www.ad.nl/ziekenhuistop100 (http://www.webcitation.org/5clUkUGcj)	Initiative of the daily paper Algemeen Dagblad (AD), in collaboration with health care professionals and medical associations. The aim is to inform the public about hospital performances. The information is generated by the paper: hospitals are asked to provide the information.
	19. Elsevier Beste Ziekenhuizen	http://www.elsevier.nl/artimg/200709/besteziekenhuizen.pdf (http://www.webcitation.org/5clUqCXSH)	Initiative of the weekly magazine, Elsevier, in collaboration with health care professionals, managers, and researchers. The aim is to inform the public about hospital performance concerning current questions in health care.
	20. Vaatpatient	http://www.vaatpatient.nl (http://www.webcitation.org/5clUrNSEI)	Initiative of the Vereniging van Vaatpatienten (VVVP) (vascular disease patient association). The aim is to support patients in their health care choices. The information is generated by external parties. The VVVP provides quality marks based on the information.

**Norway**			
	21. Fritt Sykehusvalg Norge	http://www.frittsykehusvalg.no (http://www.webcitation.org/5clV91h05)	Initiative of the Norwegian Ministry of Health in collaboration with patient advisors. The aim is to empower consumers and to support consumers and health care professionals in their choices. In addition, the aim is to stimulate competition and quality improvement.

**United Kingdom**			
	22. Dr. Foster	http://www.drfoster.co.uk (http://www.webcitation.org/5clV0liGA)	Private initiative in collaboration with the Information Centre for Health and Social Care, health service organizations, and local authorities. The aims are to inform consumers and health care professionals about the options in health care, and to support consumers in their health care choices. In addition, the aim is to stimulate quality improvement initiatives. The information is generated from a number of external sources.
	23. NHS choices	http://www.nhsdirect.nhs.uk (http://www.webcitation.org/5clVBVDFE)	Initiative of the NHS (National Health Services), in collaboration with the National Library for Health, the Information Centre for Health and Social Care, the Health care Commission and other parties. The aim is to support consumers in their decisions about health and health care.
	24. Human Fertilisation and Embryology Authority, clinics guide	http://www.hfea.gov.uk (http://www.webcitation.org/5clVXndpE)	Initiative of the Human Fertilisation and Embryology Authority (HFEA). The aims are to inform consumers about the options in health care and to support them in their health care choices. The information is generated by the HFEA and provided by the clinics.
	25. British Association of Aesthetic Plastic Surgeons	http://www.baaps.org.uk (http://www.webcitation.org/5clVJK6mK)	Initiative of the British Association of Aesthetic Plastic Surgeons. The aims are to inform the public about the practice and quality of plastic surgery and to support consumers in their health care choices.
	26. Private Healthcare UK	http://www.privatehealth.co.uk (http://www.webcitation.org/5clVKQVND)	Initiative of Intuition Communication Ltd (a commercial organization). The aims are to inform consumers about options in private health care and to support them in their health care choices.

**United States**			

	27. Hospital Compare	http://www.hospitalcompare.hhs.gov (http://www.webcitation.org/5clZSIjyU)	Initiative of the US Department of Health and Human Services (HHS). Hospital Compare is a collaboration of the Centers for Medicare and Medicaid Services (CMS), the Department of Health and Human Services, and members of the Hospital Quality Alliance (HQA). The aim is to support consumers in their health care choices. The information is provided by the health care providers.
	28. The Leapfrog Group	http://www.leapfroggroup.org (http://www.webcitation.org/5clZVjgKj)	Initiative of the Leapfrog Group (a collaboration of employers). The aim is to stimulate transparency and access to information in order to support health purchasers and consumers in their choices. In addition, the aim is to stimulate quality improvement initiatives. The information is provided by the health care providers.
	29. The Patient Advocate	http://www.opa.ca.gov/report_card (http://www.webcitation.org/5clZX6PqW)	Initiative of the Office of the Patient Advocate (OPA) in collaboration with the Department of Managed Health Care. The aim is to inform health care consumers about their rights and about the options in health care (patient empowerment). In addition, aims are to stimulate health care transparency and to support health care purchasers and consumers in their choices. The information is generated from a number of external sources.
	30. Nursing Home Compare	http://www.medicare.gov/NHcompare (http://www.webcitation.org/5clZpsiiX)	Initiative of Medicare. The aims are to inform the public about nursing home options in Medicare and to support consumers in their choices. The information is generated by external parties and/or provided by the nursing homes.
	31. Home Health care Compare	http://www.medicare.gov/HHcompare (http://www.webcitation.org/5clZlSuKF)	Initiative of Medicare. The aims are to inform the public about home health care options in Medicare and to support consumers in their choices. The information is generated by external parties and/or provided by the home health care providers.
	32. Dialysis Facility Compare	http://www.medicare.gov/dialysis (http://www.webcitation.org/5clZvYdSV)	Initiative of Medicare. The aims are to inform the public about chronic kidney disease and dialysis, about dialysis facility options in Medicare, and to support consumers in their choices. The information is generated by external parties and/or provided by the facilities.
	33. Medicare Options Compare	http://www.medicare.gov/MPPF (http://www.webcitation.org/5cla2KXFv)	Initiative of Medicare. The aims are to inform the public about health plans options in Medicare and to support consumers in their choices. The information is generated by external parties and / or provided by the plans.
	34. U Compare Healthcare	http://www.ucomparehealthcare.com (http://www.webcitation.org/5cla68Ljp)	Initiative of About, Inc (part of the New York Times Company). The aim is to support consumers in their health care choices. The information is generated from a number of external federal sources
	35. California Nursing Home Search	http://www.calnhs.org (http://www.webcitation.org/5claA0qhi)	Initiative of the California Health care Foundation in collaboration with the Department of Social and Behavioral Sciences of the University of California. The aim is to inform the public about the options in health care. The information is generated from a number of external state and federal sources.
	36. NCQA	http://www.ncqa.org (http://www.webcitation.org/5claG27UP)	Initiative of the National Committee for Quality Assurance (NCQA). The aim is to stimulate transparency and quality improvement initiatives. In addition, the aim is to support consumers in their health care decisions. The information (based on a set of standardized measures) is generated by the NCQA.
	37. US News Health	http://health.usnews.com/sections/health (http://www.webcitation.org/5claM7ca0)	Initiative of the US News magazine (which also includes a weekly digital magazine). The aim is to inform the public about performance of hospitals (America’s best hospitals) and about health plans (America’s best health plans). The information is generated by the magazine’s editors.
	38. AHD.com	http://www.ahd.com (http://www.webcitation.org/5claNNKMz)	Initiative of the American Hospital Directory, Inc. (a private company). The aim is to inform subscribers about performances of hospitals. The information is generated by the company and extracted form a number of external sources.
	39. Health Care Choices	http://www.healthcarechoices.com (http://www.webcitation.org/5claTftLr)	Initiative of Health Care Choices (HCC) which is a not-for-profit corporation. The aims are to inform the public about the health care system and to support health care purchasers and consumers in their choices.
	40. Quality Check	http://www.qualitycheck.org (http://www.webcitation.org/5claYPkbV)	Initiative of the Joint Commission on Accreditation of Health care Organizations (JCAHQ), which is a non-for-profit organization. The aim is to support consumers in their health care choices. The information is provided by the health care providers to the Joint Commission.
	41. PHC4	http://www.phc4.org (http://www.webcitation.org/5ndQxiDQX)	Initiative of the Pennsylvania’s Health Care Cost Containment Council. The aim is to increase transparency and competition between health care providers. The information is generated from hospitals and health plans by the Council.
**Sweden**			
	42. Aldre-guiden	http://www.socialstyrelsen.se/aldreguiden (http://www.webcitation.org/5cladHprj)	Initiative of Socialstyrelsen (a governmental organization of the Ministry of Health). The aims are to inform consumers about the options in elderly care and to support their choices. In addition, the aim is to stimulate quality improvement initiatives. The information is provided by local authorities.

^a^Description based on website content in September 2008.

^b^Because website content and presentation formats change over time, the URLs have been archived: the URLs within brackets can be used to view the information on the home page.

**Figure 1:**
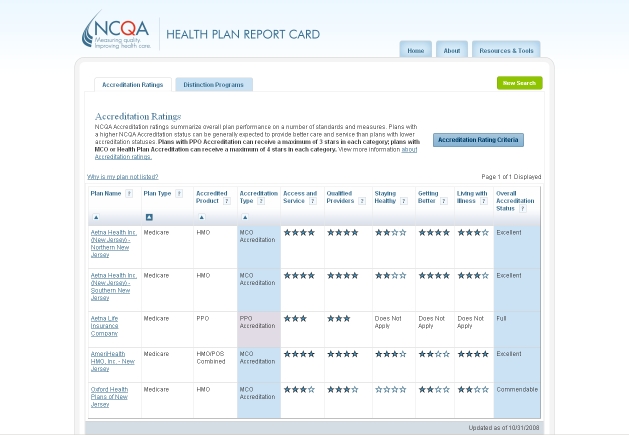
Example of a typical tabular format displaying providers in rows and attributes in columns

Most websites contained, in one way or another, both summary and more detailed information. Summary information was usually presented in tabular formats using rows to display providers and columns to display attributes (see Figure 1). Tables with a display configured differently (ie, providers in columns and attributes in rows) were also common but this configuration was not typically used in summary tables. However, tables configured in this way were frequently found to present more detailed comparative information pertaining to the specific providers selected. Although some summary tables presented many different attributes, in most cases only a limited number of attributes (about 3 to 7) was displayed. In some summary tables, the main attributes were divided into subattributes. Another frequently used method was to allow the consumer to determine the amount of information to be presented in a table.

### Information Characteristics

Table 2 provides an overview of the information characteristics on the reviewed websites.

**Table 2:** Reviewed websites and their information characteristics

Website^a^	Health Care Sector	Types of Information^b^	Classification of Integration Format^c^	Drill Down Paths	Information Display	Rationale for Presentation Formats^d^
1. Your Hospitals	Hospitals	B, C	2	No drill down paths, reports downloaded as PDF files	Words; numbers	-
2. Hospital Report	Hospitals	B, C	0	No drill down paths, reports downloaded as PDF files	Numbers	-
3. Sundhed	Hospitals	A, B, C	0 (separate pages for different types of information); 2 (different types in one table by consumer choice)	Drill down paths to same information per provider	Numbers; stars (5); capitals	-
4. Weisse Liste	Hospitals (will include nursing homes and rehabilitation facilities in near future)	A, B, C	1	Drill down paths to more specific information per hospital	Words; numbers; horizontal bars; round icons (favorites)	D, E, F
5. Klinik Führer Rhein-Ruhr	Hospitals	A, B, C	3	Drill down paths to more specific information per hospital	Words; numbers; thermometers	-
6. Klinikführer Rheinland	Hospitals	A, B	3	Drill down paths to more specific information per hospital	Words; numbers; traffic lights (3 colors); horizontal bars	D, E, F
7. Hamburger Krankenhaus-spiegel	Hospitals	A, B	0	No drill down paths	Numbers; horizontal bars	-
8.Klinikbe- wertungen	Hospitals	A, C (anecdotal information)	3	Drill down paths to specific evaluations of patients	Numbers; stars (6); words	-
9. Health Information and Quality	Hospitals	B	-	No drill down paths, reports downloaded as PDF files	Words in different colors (= symbols)	-
10. kiesBeter	Hospitals, nursing homes, home care, outpatient mental health care, care for the handicapped, primary care, palliative care, health plans	A, B, C	0 and 2 (depending on health care sector); 3 (summary information)	Drill down paths to more detailed information	Words; numbers; capital letters; stars (3); stars (5); horizontal bars (1)	D, E, F
11. Independer Gezondheids-zorg	Hospitals, home care, primary care, physiotherapy, health plans	A, B, C	3	Drill down paths to more specific information per provider	Words, numbers, stars (4), stars (5), round icons (colored), coins, horizontal bars	D, E, G
12. Zorgkiezer	Hospitals, health plans	A, B	3	Drill down paths to more specific information per provider	Words, numbers, stars (5), checkmarks	-
13. Zorgbelang	Nursing homes, home care, care for the handicapped, outpatient mental health care	A (links to websites with B and C)	-	No drill down paths	Words	-
14. Agis Zorggids	Contracted hospitals	A ,C	0	No drill down paths	Words, round icons (3)	E, F
15. Menzis behandelwijzer	Contracted hospitals	A, B, C	3	No drill down paths	Words, numbers, stars (4), round icons (with certain degree of filling), plus icons (3)	F, G
16. VGZ Zorggids -Vergelijk en kies	Contracted hospitals and other providers	A, B, C	3	No drill down paths	Words, numbers, squares (4)	-
17. CZ Ziekenhuisver-gelijker	Contracted hospitals	A, B, C	1	No drill down paths	Words, numbers, stars (4), stars (5)	-
18. AD Ziekenhuisver-gelijker	Hospitals	B, C	2	Drill down paths to more specific information per provider	Words, numbers	-
19. Elsevier Beste Ziekenhuizen	Hospitals	A, B	3	No drill down paths, reports downloaded as PDF files	Round icons (5, colored), horizontal bars	-
20. Vaatpatient	Hospitals	A, B	2	Drill down paths to more specific information per provider	Numbers, checkmarks	-
21. Fritt Sykehusvalg Norge	Hospitals	A, B, C	1	Drill down paths to somewhat more detailed quality information	Numbers, words, symbols (-, +, 0)	-
22. Dr. Foster	Hospitals, specialized clinics, complementary practitioners	A, B	1 (with exception of distance)	Drill down paths to more specific information per hospital; selection options to obtain more detailed information	Words,numbers, horizontal bars, stars (5), squares (3)	-

23. NHS choices	Hospitals	A, B, C	3 (summary information); 1 (detailed information)	Drill down paths to more detailed information; drill down paths to more specific information per provider	Words, numbers, round icons with words, stars (3), horizontal bars, squares (5)	-
24. Human Fertilisation and Embryology Authority, clinics guide	Specialized clinics	A, B	0	No drill down paths	Words, numbers, horizontal bars, triangles (1)	-
25. British Association of Aesthetic Plastic Surgeons	Plastic surgeons	A, B	2	Drill down paths to more specific information per provider	Words, numbers, stars (1)	-
26. Private Healthcare UK	Hospitals, doctors, GP’s, nursing homes, cosmetic surgery, dental care, health plans	A, B, C (anecdotal information)	1	Drill down paths to more specific information per provider	Words, numbers, ribbons (1)	D

27. Hospital Compare	Hospitals	A, B, C	3 (summary information); 2 (after selection of hospitals)	Drill down paths to hospital location on map	Words, numbers, horizontal bars (1)	-

28. The Leapfrog Group	Hospitals	B	-	Drill down paths to more specific information per provider	Vertical bars (4), horizontal bars (1)	-
29. The Patient Advocate	Medical groups, hospitals, health plans	B, C	3 (summary information); 0 (detailed information)	Drill down paths to more detailed information	numbers, stars (4), horizontal bars (1), round icons with words and colors (5)	-
30. Nursing Home Compare	Nursing homes	A, B	0 (summary information); 2 (detailed information)	Drill down paths to more specific information per provider; drill down paths to provider location on map; drill down paths to visual display in bar graphs	Words, numbers, cubes in bar (4), horizontal bars	-
31. Home Health care Compare	Home care	A, B	0 (summary information); 2 (detailed information)	Drill down paths to visual display in bar graphs	Words, numbers, checkmarks, horizontal bars	-
32. Dialysis Facility Compare	Specialized centra	A, B	0 (summary information); 1 (detailed information)	Drill down paths to more specific information per provider; drill down paths to more detailed quality information; drill down paths to provider location on map	Words, numbers, horizontal bars, checkmarks	-
33. Medicare Options Compare	Health plans	A, B, C	0 (summary information); 3 (detailed information)	Drill down paths to more specific information per health plan	Words, numbers, stars (5)	-
34. U Compare Healthcare	Doctors, hospitals, nursing homes, health plans, mammography centers; fertility clinics	A, B	1	Drill down paths to more specific information per provider	Words, numbers, vertical bars (1), plus icons (1), checkmarks	-

35. California Nursing Home Search	Nursing homes, home care, hospices	A, B	3 (summary information); 1 (detailed information)	Drill down paths to more specific information per provider	Words, numbers, stars (3)	D
36. NCQA	Doctors, health plans	A, B, C	3 (summary and detailed information)	Drill down paths to more detailed quality information; drill down paths to more specific information per provider	Words, numbers, stars (4), horizontal bars, certification symbols (1)	-
37. US News Health	Hospitals, health plans	B, C	0 (hospitals); 3 (health plans)	Drill down paths to more detailed information; drill down paths to more specific information per provider	Words, numbers, round colored icons (5), stars (5)	-
38. AHD.com	Hospitals	A, (B and C only when for members)	0 (summary and detailed information)	Drill down paths to more specific information per provider	Words, numbers, colored parts	-
39. Health Care Choices	Hospitals (and doctors for pay)	A, links to B	-	No drill down paths	Words, numbers	-

40. Quality Check	Hospitals, nursing homes, home care, outpatient mental health care	A, B	1 (summary information); 0 (detailed information)	Drill down paths to more specific information per provider; drill down paths to more detailed information	Words, numbers, checkmarks, certification symbols (1), round icons (3)	-
41. PHC4	Hospitals, health plans	A, B, C	3 (hospitals); 0 (health plans)	No drill down paths, reports downloaded as PDF files	Numbers, round icons (3)	F
42. Aldre- guiden	Care for the elderly	B	-	Drill down paths to more detailed quality information	Numbers, vertical bars	D, E, F

^a^Description based on website content in September 2008. Website content and presentation formats change over time. Therefore, the URLs have been archived (see Table 1).

^b^This classification is based on Van Loon and Tolboom [20]: A = Factual information based on provider characteristics and services; B = Quality information based on performance indicators; C = Quality information based on health care user experience

^c^0 = no integration of different types of information (different types of information on different pages); 1 = limited integration of different types of information (different types of information can be selected and viewed on one page, but no integration in one table on one page); 2 = quite amount of integration of different types of information (different types on one page, but clearly separated from each other); 3 = high level of integration of different types of information (different types of information presented in one table, with or without action of the consumer).

^d^D = test(s) of different formats; E = existing scientific knowledge; F = expert opinion; G = other rationale

### Health Care Sectors

On 32 of the 42 websites (76%), information about hospitals was presented. Although in recent years more information has become available in other health care sectors, such as nursing homes and home care (found on 10 websites; 24%), and health plans (found on 10 websites; 24%), hospital information clearly had the largest share on the Internet. Information about health plans was found mainly on US websites. Reporting systems containing information on several health care sectors were found mainly on websites from the United Kingdom and the United States.

### Types of Information

The most common type of information found on the reviewed websites was quality information based on performance indicators (found on 37 websites; 88%). Information on health care providers’ characteristics and services was also common (found on 34 websites; 81%); this information was usually presented for each provider separately. In these cases, we did not further evaluate the information. Quality information based on health care users’ experiences was found on a little more than half (found on 22 websites; 52%) of the reviewed websites.

### Integration of Different Information Types

The degree of integration of different information types was most often classified as type 0 (no integration of different types of information; different types of information on different pages). This type of information integration was found on 15 websites (36%). Type 3 (high level of integration of different information types; different types of information presented in one table) was found on 17 websites (41%). The two integration structures falling in between these extremes were less often found: type 1 on 10 websites (24%) and type 2 on 9 websites (21%), respectively. Concerning type 1 and type 2 integration, many different options were used to separate the information types. For example, separate tab pages, menu bars, white spaces, bold headlines, and colors to distinguish between different information types were displayed. In some cases, different information displays were used at the same time.

**Figure 2:**
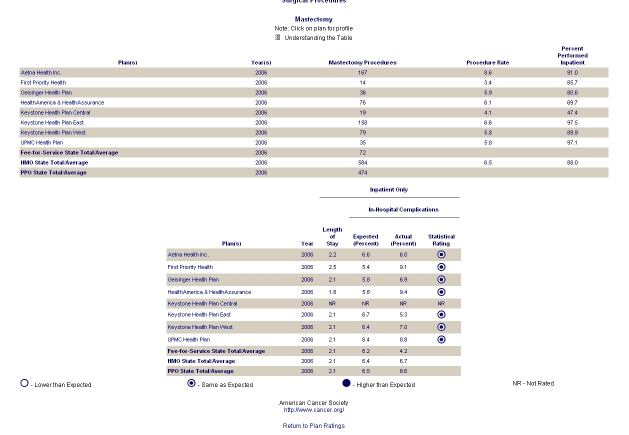
Example of type 0 integration (no integration of different types of comparative information)

**Figure 3:**
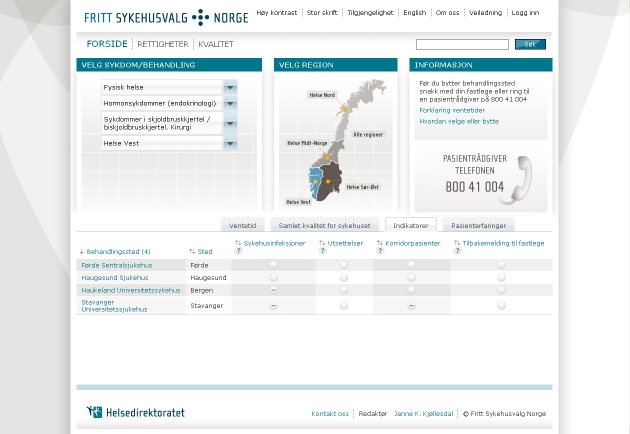
Example of type 1 integration (limited integration of different types of comparative information)

**Figure 4:**
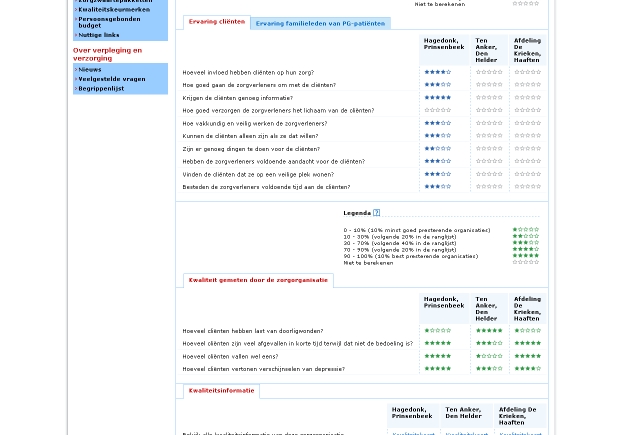
Example of type 2 integration (medium amount of integration of different types of comparative information)

**Figure 5:**
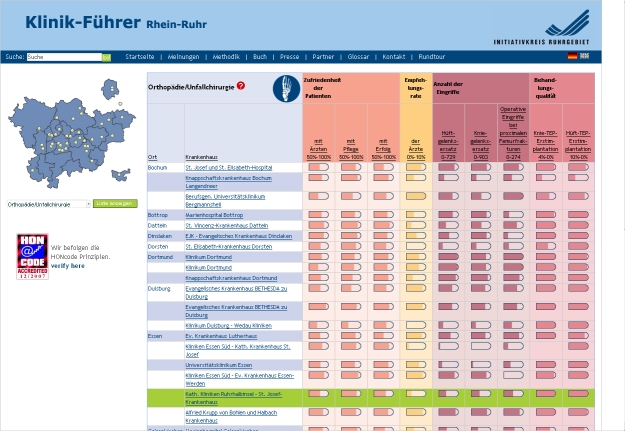
Example of type 3 integration (high integration of different types of comparative information)

Examples of all four classifications are shown in Figures 2 to 5. Figure 2 is an example of type 0 integration (no integration of different types of comparative information). The example is from the PHC4 website in the United States. In this example, information on health care user experience is displayed, but information based on performance indicators can be found elsewhere on the website. Figure 3 is an example of type 1 integration (limited integration of different types of comparative information). The example is from the Fritt Sykehusvalg website in Norway. In this example, different information types can be selected on the displayed tab pages, but are not displayed in a single table simultaneously. Figure 4 is an example of type 2 integration (a medium amount of integration of different types of comparative information). This example is from the kiesBeter website in the Netherlands. Different information types on one page are presented in separate blocks. Figure 5 is an example of type 3 integration (high integration of different types of comparative information). This example is from the Kliniken Rhein Ruhr website in Germany. Different information types are integrated in a single table.

### Drill Down Paths

**Figure 6:**
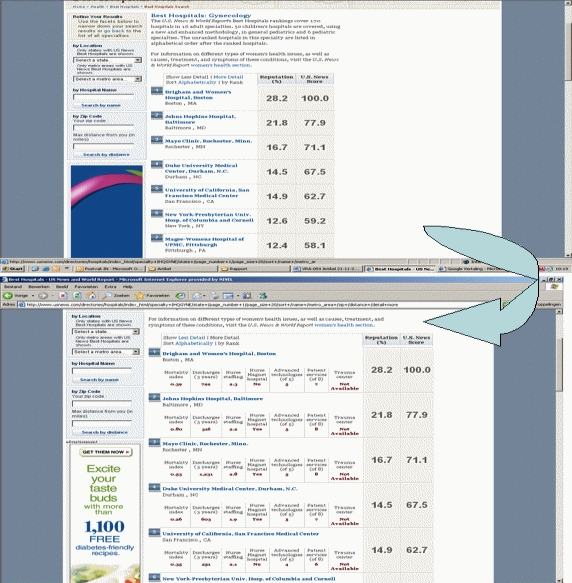
Example of drill down path to more detailed information

A considerable number of websites (29; 69%) provided drill down paths to more specific information. The most common types of drill down paths were paths to more specific information per provider (on 21 websites; 50%) and paths to more detailed (underlying) information (on 11 websites; 26%). The information per provider to which a Web page was linked usually consisted of very specific information listed on a single Web page. Concerning more detailed comparative information, the degree to which more specific information was provided differed across websites. Figure 6 shows an example of more detailed information available after drilling down. The example is from the website US News Health. In this example, more detailed information can be found by clicking on “more detail.”

### Information Display

To display comparative health care information, numbers (37; 88%) and words (32; 76%) were most commonly used. Most often, information about provider characteristics and services was presented by using words and numbers only. Graphical formats and symbols were frequently applied as well, usually to present quality information. The most frequently applied symbols were stars (on 15 websites; 36%; see Figures 1 and 4) and round icons (on 10 websites; 24%; see Figure 2). The numbers of stars, round icons and other symbols differed both across and within websites: five, four, and three symbols were most frequently found. Furthermore, it was quite common (on 18 websites; 43%) to use bar charts to present quality information.

### Rationale for Presentation Formats

In total, 10 of the 42 websites (24%) returned a completed survey. Of these 10, the most common rationales for the presentation formats used were expert opinion and tests with consumers and/or other stakeholders (both found on 7 websites; 70% of the responding websites) (see Table 2).

## Discussion

### Principal Results

We reviewed 42 websites providing public comparative health care information and analyzed the presentation approaches of different information types. The general conclusion is that a wide variety of presentation approaches are used on Web-based reporting systems, in particular with respect to the integration of different information types and the information display. The two extreme options to integrate different information types were most often found: providing no integrated information at all and presenting a high level of integration in a single table. Between these two extremes, different options to either separate or integrate the information types were applied. Although different presentation formats were found, some standard elements emerged as well. On most websites, for example, tabular formats were used that presented providers in rows and indicators in columns. The majority of information was provided hierarchically, with options to get an overall sense of performance provided first, and options to get more detailed information provided subsequently. This format seemed necessary to manage the total amount of available information.

### Study Limitations

Our study was intended to provide an impression of existing presentation approaches of comparative health care information. Clearly, not all aspects related to information presentation have been systematically reviewed. Although it is beyond the scope of the current study, it is important to keep in mind that the quality of the information itself has not been assessed. Websites may vary on the quality of the information collected and presented. We believe, however, that the current study results provide insight into the state of the art concerning the presentation of comparative health care information in the late 2000s. Our study might be limited by the fact that the search strategies were performed solely by native speakers of Dutch. The number of websites per country might be biased toward including more Dutch websites. And, in general, the number of websites found per country may be partly influenced by each author’s mastery of the different languages included in the search. We only captured Western websites, and the results should therefore be interpreted as only representative of Western websites. Another limitation is the fact that the response rate of the survey was very low. Because of this low response rate, we had limited insight into how information was tested and what consumers’ reactions were. From the returned surveys, it appeared that consulting experts and tests with consumers were important methods to select presentation formats. It is unknown whether these methods are representative of those used for development of the other websites included in the study.

### Conclusions

Regarding the usefulness of comparative information for consumers, several results related to the reviewed presentation formats are worth discussing further. 

First, the standard use of tabular formats to structure the information is important. On the investigated websites, the use of rows for providers and columns for attributes was the typical format for displaying summary information, whereas the opposite display format was used for more detailed information (after selection or drill down paths). It would be relevant to determine whether it makes a difference for consumers to see either providers or attributes in rows. It is known that consumers use both holistic processing (providers first) and dimensional processing (attributes first) with a slight preference for the latter [34]. Swait and Adamowicz [23] argued that the more complex information is, the simpler the heuristics that are used, which results in readers focusing more on alternatives (providers) than on attributes. From these findings we conclude that it is not the direction of the information display that is particularly important, but rather the information complexity in the table. Given the fact that most consumers will probably view only summary information, these tables should thus contain graspable numbers of providers and attributes. Otherwise, consumers will not concentrate on the attribute information even though this is the information that has been provided to support their decisions.

A second important aspect to consider is the variety of information display options found on websites. Words as well as numbers were frequently used to present comparative information. It is striking that numbers were displayed on so many websites although it is known that consumers have difficulty evaluating them [21]. As recently demonstrated by Peters and colleagues [35], numbers do not have evaluative meaning to consumers. On a large number of the websites, however, information was presented using symbols. Hibbard and colleagues [21,27] argued that visual cues such as stars increase the evaluability of information, because these cues help consumers sort providers into categories of better and worse. Furthermore, symbols might more easily attract attention compared with numbers and words, similar to pictorial information [36,37]. Pictures seem to promote a more holistic and integrative strategy to process information than do words [38]. However, when there is text-symbol incongruity, symbols may decrease message comprehension, especially among consumers having low literacy [39]. In an experiment by So and Smith [24], symbols (smiley faces) added to tabular information did not facilitate consumers’ decision accuracy. Future research on comparative health care information should include similar experiments and examine the impact of symbols. The use of stars,which were frequently found on the reviewed websites, may be an effective presentation format of comparative health care information. More research is needed to confirm this.

Third, attention should be focused on the integrated presentation of different information types. To our knowledge, there are no studies that examined the effects of integration levels of different information types. Hence, we cannot make scientifically based inferences about how the different degrees of integration found on the websites included either support or impede consumer decision making. Compared with the 1996 review of McCormack et al [25], who analyzed the content of comparative health care information, it is important that more “objective” performance indicators are dominant in the current review (included in 88% of the reviewed websites). In the findings of McCormack et al, such performance indicators were included in 10 out of 24 (24%) reporting systems, all in combination with health care user experience data. Despite the lack of evidence for consumer reactions, some arguments about the advantages and disadvantages of integrating information types can be made. One important benefit of a high level of integration is that all information can be viewed in an overview at the same time. This may contribute to a sense of clarity and to better coping with a large amount of information. A drawback is that such an overview cannot take up too much space on Web pages, and that the chance that a page will contain contradictory information increases. In addition, more specific information will be lost or difficult to find for consumers, and the flexibility to apply different search strategies diminishes. The opposite of no or very limited integration can, however, also bring about negative consequences. For example, consumers may not see a large part of the information at all or may fail to notice important information elements. In addition, consumers may need to undertake many steps in the process of viewing information, although it is known that consumers prefer to see information on one Web page [40]. An approach advocated by Harris-Kojetin et al [19] is to help consumers to think about their own priorities in the major dimensions of health care. This approach using self-selection menus could be applied to assess whether consumers are more focused on technical outcomes of health care or more focused on aspects related to trust in health care. The fact that these two health care consumer profiles can be distinguished among different patient groups [41] may be used as argument for low levels of integration of different information types. However, the approach of self-testing consumer preferences assumes that consumers have stable preferences, although we know that consumers often construct these preferences while viewing information [42]. All in all, the issue of integrating different information types remains an important topic for further discussion and, importantly, for future research on health care information. In our opinion, a certain level of integration is necessary to prevent consumers overlooking important information or getting stuck in too many decision steps.

A fourth topic for further discussion is the role of contradictory information, which appears to be inherent in comparative health care information. As stated, a higher integration of different information types increases the chance that contradictory information must be processed. It is usually assumed that conflicting information increases task complexity. Psychological theories such as cognitive dissonance theory [43] suggest that when people meet aspects of their decision environment that are incompatible with each other, they attempt to reestablish consistency by transforming some of the incompatible elements. The activities associated with this restoring process are known to demand elaboration [44], and will probably lead to distress as well. Individuals tend to avoid conflict or to avoid choosing at all when choices become more complex [14,45-48]. In addition, there is a higher chance individuals will use simpler choice heuristics [23]. At this time, it remains unclear how to deal with the issue of contradictory information. It is important that future studies search for comprehensible presentation formats that facilitate correct processing of contradictory information. Meanwhile, website managers should be careful not to present information that includes many contradictory elements.

Finally, we want to address the large amount of information we found on websites. It is known that today’s consumers are often overloaded with information. Different effects of information overload have been described in the literature. Importantly, a large amount of information can lead to low quality of consumers’ choices [14] and to less purchasing [49]. Lurie [50] showed that the amount of information that needs to be processed not only depends on the number of alternatives and attributes in a choice set, but also on the number of attribute levels and the distribution of attribute levels across alternatives. To control the amount of information on websites, it seems necessary to provide only limited numbers of providers and attributes to consumers, as was already suggested concerning information complexity. When a large variety of attribute levels are shown, Web designers and research staff should note the increasing complexity and search for alternative options to display information. Drill down paths can be used to layer information and to comprehensibly provide a large amount of information, as was done on many websites reviewed in this study. Furthermore, it may be necessary to inform consumers on the home page about the amount of information that can be viewed on the website. Consumers will then be better prepared and perhaps less discouraged when they attempt to access the information. Future research should focus on the amount of information that consumers are able and willing to process.

With the current descriptive study, we have shed some light on the decision environment of health care consumers in a period of market-based, consumer choice-driven health care sectors. We believe that more transparency about the effectiveness of the chosen formats on websites is greatly needed; currently it is largely unclear which rationales are used to select them. Evidence-based quality criteria for presentation approaches should be formulated, and future research can assess how different websites meet these criteria. Moreover, research is needed on other aspects of the decision environment, such as consumers’ considerations and motivations to achieve a (good) decision and their decision strategies. Consumers highly motivated to search for good performance might be less distressed by complex information presentation than people who do not care to actively choose health care in any case. More generally, the design of websites should be linked to theoretical models of consumer decision making and communication technology. In our opinion, it is a challenge for Internet research to create more manageable comparative health care information that is actually used by consumers. Current presentation approaches on websites do not seem to be systematically selected. Website managers should not just release data on the web, but instead should become aware of the many complexities inherent in the comparative information they are providing.
